# Supplementary material for: EnzML: multi-label prediction of enzyme classes using InterPro signatures
Source: BMC Bioinformatics. 2012 Apr 25;13:61. doi: 10.1186/1471-2105-13-61 (PMC3483700; doi:10.1186/1471-2105-13-61)
Supplement: Addtional file 5 — The Java code to format the data files, evaluate and predict. The file enzml_java_code.tar.gz contains the Java code used to format database data to ARFF and XML formats, to execute cross and train-test (jackknife) evaluations and to record evaluation results to database. More information is included in the readme.txt file and the Javadoc files. The code can be used with a MySQL database. To use a different database software, other JDBC drivers might be required. [file 1471-2105-13-61-S5.gz › java_code/utils/doc/edu/cornell/lassp/houle/RngPack/class-use/RandomElement.html]

Uses of Class edu.cornell.lassp.houle.RngPack.RandomElement


---


|  |  |  |
| --- | --- | --- |
| **Overview** \| **Package** \| **Class** \| **Use** \| **Tree** \| **Deprecated** \| **Index** \| **Help** | |  |
| PREV   NEXT | **FRAMES**    **NO FRAMES**     **All Classes** |  |


---


## **Uses of Class edu.cornell.lassp.houle.RngPack.RandomElement**

| Packages that use RandomElement | |
| --- | --- |
| **cern.jet.random** |  |
| **cern.jet.random.engine** |  |
| **edu.cornell.lassp.houle.RngPack** |  |

Uses of RandomElement in cern.jet.random

| Methods in cern.jet.random that return RandomElement | |
| --- | --- |
| `static RandomElement` | `AbstractDistribution.makeDefaultGenerator()`             Constructs and returns a new uniform random number generation engine seeded with the current time. |

Constructors in cern.jet.random with parameters of type RandomElement

`Pareto(double k,
double alpha,
RandomElement randomGenerator)`
  
          Constructs a Pareto distribution.

Uses of RandomElement in cern.jet.random.engine

| Subclasses of RandomElement in cern.jet.random.engine | |
| --- | --- |
| `class` | `MersenneTwister`             MersenneTwister (MT19937) is one of the strongest uniform pseudo-random number generators known so far; at the same time it is quick. |
| `class` | `RandomEngine`             Abstract base class for uniform pseudo-random number generating engines. |

| Methods in cern.jet.random.engine that return RandomElement | |
| --- | --- |
| `static RandomElement` | `RandomEngine.makeDefault()`             Constructs and returns a new uniform random number engine seeded with the current time. |

Uses of RandomElement in edu.cornell.lassp.houle.RngPack

| Subclasses of RandomElement in edu.cornell.lassp.houle.RngPack | |
| --- | --- |
| `class` | `RandomJava`             RandomJava is a class wrapper for the `Math.random()` generator that comes with Java. |
| `class` | `RandomSeedable`             `RandomSeedable` is an abstract class that extends the `RandomElement` class to include the ability to automatically generate a valid `long` seed from the clock. |
| `class` | `RandomShuffle`             RandomShuffle uses one random number generator to shuffle the numbers produced by another to obliterate sequential correlations. |
| `class` | `Ranecu`             Ranecu is an advanced multiplicative linear congruential random number generator with a period of aproximately 10^18^. |
| `class` | `Ranlux`             RANLUX is an advanced pseudo-random number generator based on the RCARRY algorithm proposed in 1991 by Marsaglia and Zaman. |
| `class` | `Ranmar`             RANMAR is a lagged Fibonacci generator proposed by Marsaglia and Zaman and is a good research grade generator. |

Constructors in edu.cornell.lassp.houle.RngPack with parameters of type RandomElement

`RandomShuffle(RandomElement ga,
RandomElement gb,
int ds)`

---


|  |  |  |
| --- | --- | --- |
| **Overview** \| **Package** \| **Class** \| **Use** \| **Tree** \| **Deprecated** \| **Index** \| **Help** | |  |
| PREV   NEXT | **FRAMES**    **NO FRAMES**     **All Classes** |  |


---
